# Giant inguinal hernia repair using standard transverse inguinal incision with mesh. A retrospective case control study

**DOI:** 10.1186/s12893-023-02084-6

**Published:** 2023-06-27

**Authors:** Jonathan Abraham Demma, Rachel Gefen, Ofek Shpigelman, Alon Pikarsky, Gidon Almogy

**Affiliations:** 1 Department of General Surgery and Traumatology, Hadassah Medical Center and Faculty of Medicine, Hadassah Hebrew University Medical Center, Kalman Ya’akov Man St, Jerusalem, Israel; 2 Faculty of Medicine, Hebrew University of Jerusalem, Jerusalem, Israel

**Keywords:** Giant inguinal hernia, Tension-free repair, Abdominal compartment syndrome

## Abstract

**Background:**

Giant inguinal hernia (GIH) is a rare condition in the developed world, and the literature is scarce. Case reports describe different techniques in an attempt to prevent abdominal compartment syndrome (ACS). We aimed to review our experience with GIH repair.

**Method:**

A retrospective review of the medical records of all consecutive patients who underwent a tension-free mesh GIH repair using a transverse inguinal incision between 2014 and 2021 at a tertiary university referral center. In brief, the technique included head-down positioning, maximal pre-incision reduction of hernia contents, and repair with mesh. Follow-up was conducted in outpatient clinic. We compared the results to a time-based open standard inguinal hernia repair group (control group).

**Results:**

During the study period, 58 patients underwent an open GIH repair with mesh without abdominal preparation. 232 patients were included in the control group. The mean surgery duration was 125.5 min in the GIH group and 84 min in the control group (p < 0.001). Bowel resection was not necessary in any case. In-hospital complication rates were 13.8% vs. 5.6% in the GIH and control groups, respectively (p = 0.045). Early complication rates (up to 30 days post-operatively) were 62.1% vs. 14.7% in the GIH and control groups, respectively (p < 0.001). Late complications rate was similar (p = 0.476). ACS and mortality were not reported. No recurrence event was reported in the GIH group.

**Conclusion:**

Tension-free mesh repair for GIH using a standard transverse inguinal incision is feasible and safe and there is no need for abdominal cavity preparation. Early complications are more common than in the control group, but there were no higher rate of late or severe complications and no recurrence event.

## Introduction

Inguinal hernia is a common situation and one of the most common surgical procedures [1, 2]. A giant inguinoscrotal hernia is defined as an inguinal hernia extending below the midpoint of the inner thigh when the patient is standing [3]. Giant inguinoscrotal hernia (GIH) is a rare condition in developed countries [4, 5] and is often associated with neglect and refusal to admit to the problem [5]. GIH affects a patient’s quality of life and can result in difficulty in daily activities such as walking and voiding [3, 5, 6], along with cutaneous complications, interruption in sexual function, pain, and the risk of strangulation. When GIH persists, loss of domain can occur. The concerns that emerge in the surgical treatment of GIH differ from those for a ‘standard’ inguinal hernia. Along with a high recurrence rate [7], the main concern for GIH repair is the development of abdominal compartment syndrome (ACS), which means dangerously elevated intra-abdominal pressure. This can further result in respiratory decompensation, decreased venous return, and dangerous reduction in organ perfusion, leading to organ failure [4, 6]. Different approaches have been suggested to avoid ACS, including preoperative progressive pneumoperitoneum administration, organ resection during surgery, and component separation techniques. Nevertheless, literature regarding surgical outcomes after GIH repair is scarce and consists mostly of case reports and experimental series [4, 6, 7].

This comprehensive cohort study reviewed the experience of a single surgeon with GIH repair in a tertiary surgery hospital compared to a control group of patients who underwent standard open inguinal hernia repair. We hypothesized that patients who underwent GIH repair would have a higher early post-operative complication rate but no higher recurrence rate than patients with a standard hernia.

## Methods

### Study design and patient inclusion

This retrospective study was performed under the approval of the local ethics committee (HMO 21–0238); the study was conducted according to the Helsinki Declaration principle.

**Fig. 1:**
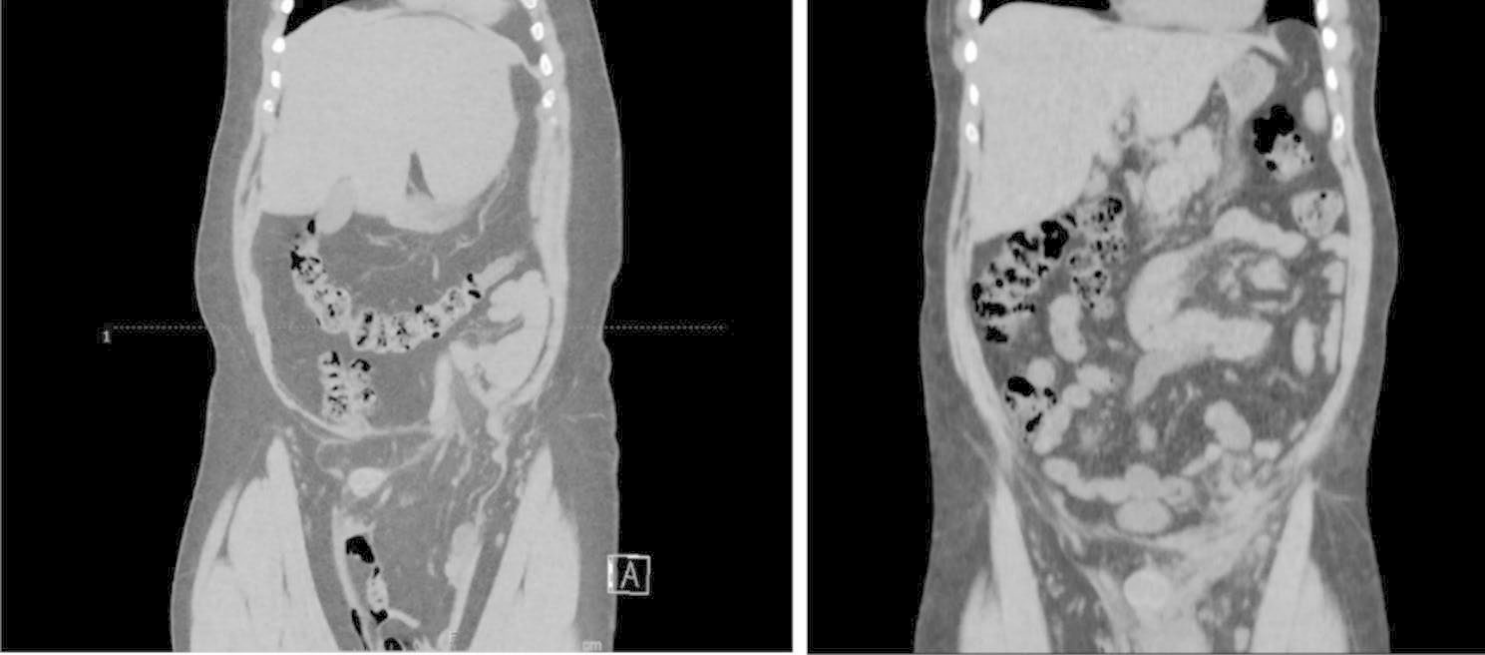
Computerized Tomography scan of a different patient with GIH before (left) and after (right) surgery

We retrospectively reviewed the charts of consecutive patients who underwent open GIH repair with mesh placement between January 1, 2014, and December 31, 2021. GIH was defined as an inguinal hernia extending below the mid-thigh in an upright position (Fig. 1). Each patient with GIH was compared to a time-based control group of patients with a standard inguinal hernia who underwent open repair in a 4:1 ratio. The same expert hernia surgeon performed all cases in both the GIH and control groups. Patients aged < 18 years, pregnant women, and those who underwent inguinal hernia repair other than in an open inguinal incision (e.g., laparoscopic repair or repair via a medial incision) were excluded. Recurrent ipsilateral hernias were included in the study.

### Surgical technique

**Fig. 2:**
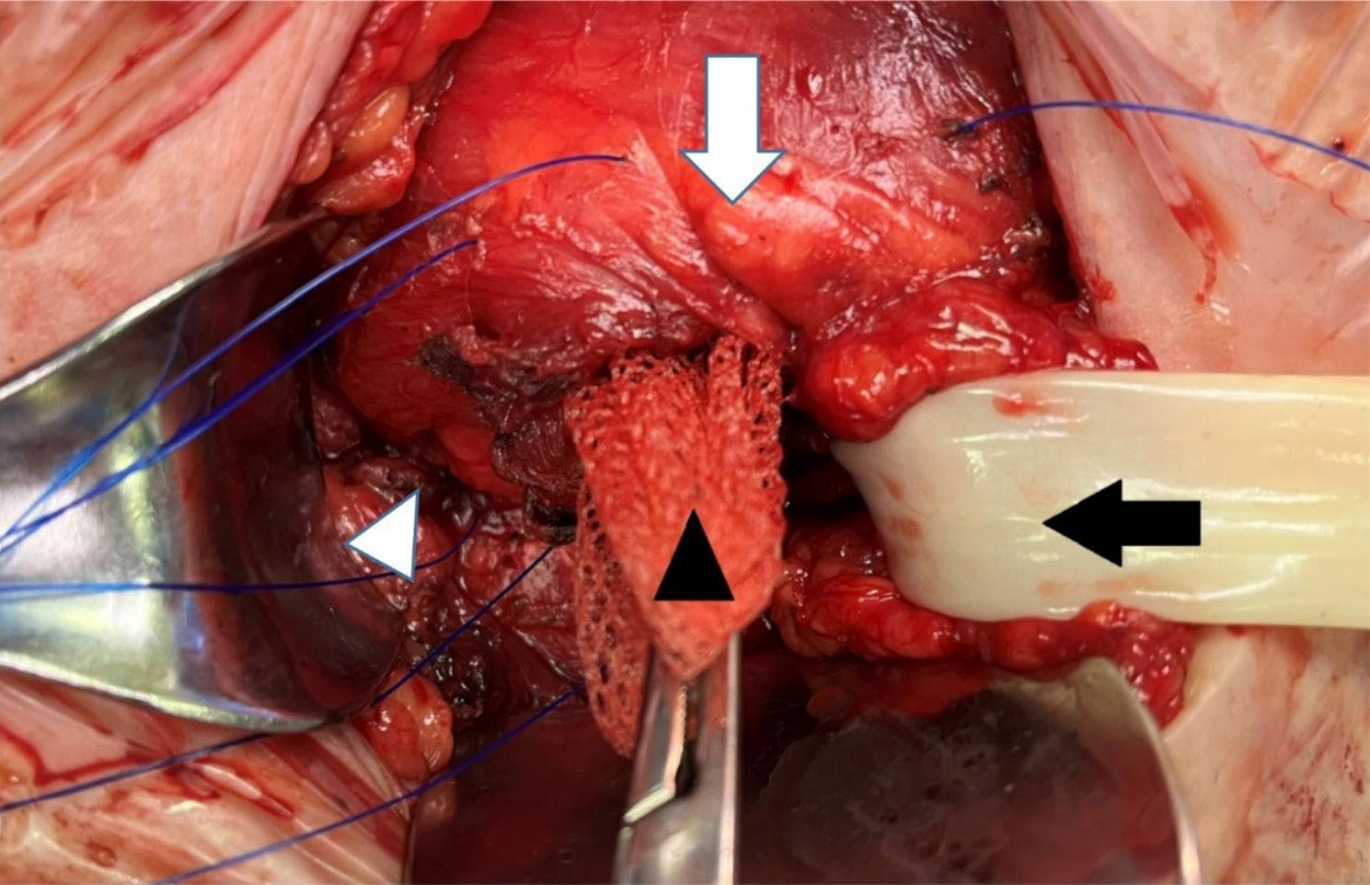
Intraoperative picture of left transverse inguinal incision during GIH repair. Black arrowhead showing a UHS mesh inserted in between the approximation sutures (conjoint tendon [white arrow] to inguinal ligament [white arrowhead]). The spermatic cord is retracted aside with a penrose drain (black arrow)

Patients with GIH underwent either general or regional anesthesia. When a urinary catheter was inserted for the procedure, it was usually removed the following morning. Patients were positioned supine in a slight head-down position (Trendelenburg position) in order to facilitate reduction of the hernia contents. Short-acting muscle relaxants were administered under general anesthesia. A manual attempt was then made to maximally reduce the hernia contents before making the incision. We performed a transverse inguinal incision, extending medially, caudally, and/or laterally as necessary. An incision was made in the external oblique fascia. At this stage, manual pressure was again applied to the scrotum to further reduce the hernia contents, while the cremasteric muscle extending into the scrotum was gradually incised. Following incision of the external fascia, more manual pressure was applied to the scrotum, usually resulting in complete reduction of the hernia contents. An attempt was made to avoid opening the hernia sac and sac resection, and complete en-bloc reduction of the sac contents was preferred. An ULTRAPRO® Hernia System (UHS) mesh was used in all cases. A tension-free approximation of the conjoint tendon to the inguinal ligament was performed in cases of large defects in order to facilitate spreading of the mesh in the pre-peritoneal space. (Fig. 2). The size of the defect was gauged by the operating surgeon during the procedure by visual evaluation of the hernia defect size and body size of the patient. A Jackson-Pratt drain was used when the resulting space created by hernia reduction was deemed large. The patients in the control group underwent open tension-free hernia repair with the same mesh under either general or regional anesthesia. Our policy is to administer prophylactic antibiotics, preferably a first generation cephalosporin, within 30 min prior to incision. Antibiotics are continued for 24 h in cases where drains are inserted. The patients were usually discharged the following day.

### Data collection and outcomes measures

The collected data included patient demographics, comorbidities, hernia side and size, and duration of hernia (defined by the patient as weeks, months, or years). The procedure parameters included anesthesia type, type of repair and mesh, urgency status, need for organ or hernia sac resection, insertion of a urinary catheter, drainage, and operation time.

Outcomes included in-hospital length of stay (LOS), complication rate, type of complication, treatment required, Clavien-Dindo grade, reoperation, follow-up, and recurrence. Complications were defined as early (within 30 days of surgery) and late (> 30 days after surgery). Early complications were further subdivided into in-hospital and post-discharge complications. Diagnosis of complications was generally based on clinical evaluation. Surgical site infection (SSI) was defined by the need to drain the wound or administer post-operative antibiotics. Diagnosis of ischemic orchitis was based on cord and/or scrotal tenderness, inflammation, and edema, without the need for imaging. Seroma and hematoma were also diagnosed clinically.

### Statistical analysis

Descriptive statistics were computed to determine frequencies and summary statistics (means, standard deviations, medians, IQR, and percentages). The χ2 test and Fisher’s exact test were used to analyze categorical variables. Student’s t-test and Mann-Whitney U test were used for continuous variables. To analyze the predictors of complications, we used a multivariate logistic regression model. Statistical significance was set at P < 0.05. Statistical analysis was performed using Software Package for Statistics and Simulations (IBM SPSS version 22, IBM Corp, Armonk, NY).

## Results

### Patients and hernia characteristics

Fifty-eight GIH repairs via inguinal incision were performed during the study period without abdominal cavity preparation. Two hundred thirty-two patients were included in the time-based control group. The mean age of all patients was 53 ± 19.2 years, and most patients were male (285, 98.3%). The two groups were similar in almost all baseline characteristics (Table 1). The retrospective data did not allow a comparison of Body Mass Index between the two groups. Patients in the GIH group were slightly heavier compared with those in the control group (81 kg vs. 75 kg, respectively, p = 0.04). They also had more congestive heart failure (p = 0.039). Patients in the GIH group had a higher percentage of left-sided hernias (35 [60.3%] vs. 103 [44.4%] in the control group, p = 0.039) and a higher rate of previous ipsilateral inguinal surgery (p = 0.004). The testis could not be palpated on physical examination in 24 patients (41.4%) in the GIH group compared with four patients (1.7%) in the control group (p < 0.001). In 40 patients (69%) with GIH, the intestine was palpable in the hernial sac compared with 36 patients (15.5%) in the control group (P < 0.001). Patients in the GIH group had a hernia for a significantly longer duration (53 patients [91.4%] had a hernia for more than a year compared with 79 patients [34.3%] in the control group; p < 0.001).

**Table 1:** Patients characteristics.

		Control Group	GIH Group	p value
**Number**		**232**	**58**	
Age, mean (SD)		52.91 (19.37)	53.34 (18.73)	0.876
Weight, median (IQR)		75.00 [68.00–85.00]	81.00 [69.50–89.00]	**0.04**
Gender (%)	Female	4 (1.7)	1 (1.7)	1
	Male	228 (98.3)	57 (98.3)	
ASA score (%)	1	90 (38.8)	25 (43.1)	0.52
	2	114 (49.1)	25 (43.1)	
	3	27 (11.6)	7 (12.1)	
	4	1 (0.4)	1 (1.7)	
Anti-platelet therapy (%)		33 (14.2)	4 (6.9)	0.186
Anticoagulation therapy (%)		10 (4.3)	4 (6.9)	0.49
CHF (%)		0 (0.0)	2 (3.4)	**0.039**
Connective tissues disease		9 (3.9)	3 (5.2)	0.712
Chronic lung disease (COPD/asthma)		12 (5.2)	3 (5.2)	1
CRF (%)		3 (1.3)	3 (5.2)	0.097
End Stage Renal failure (%)		1 (0.4)	0 ( 0.0)	1
CVA (%)		6 (2.6)	2 (3.4)	0.662
DM (%)		11 (4.7)	7 (12.1)	0.061
Hematologic disease (%)		9 (3.9)	1 (1.7)	0.693
HTN (%)		58 (25.0)	13 (22.4)	0.736
IHD (%)		24 (10.3)	4 (6.9)	0.619
Hemiplegia (polio, etc.)		2 (0.9)	1 (1.7)	0.489
PVD (%)		2 (0.9)	1 (1.7)	0.489
S/p Chemotherapy treatment (%)		8 (3.4)	2 (3.4)	1
Smoker (%)		69 (29.7)	16 (27.6)	0.872
VHD (%)		5 (2.2)	1 (1.7)	1
Hernia side (%)	Left	103 (44.4)	35 (60.3)	**0.039**
	Right	129 (55.6)	23 ( 39.7)	
Hernia duration (%)	weeks	5 (2.2)	1 (1.7)	**< 0.001**
	months	146 (63.5)	4 (6.9)	
	years	79 (34.3)	53 (91.4)	
Previous ipsilateral groin surgery (%)		24 (10.3)	15 (25.9)	**0.004**
Palpable testis on physical exam (%)		228 (98.3)	34 (58.6)	**< 0.001**
Hernia contains bowel on physical examination (%)		36 (15.5)	40 (69.0)	**< 0.001**

### Surgery

Of the 58 patients who underwent GIH repair, 12.1% of the GIH group had emergency surgery compared to 3% of the control group (P = 0.01). Urinary catheters were inserted in 11 (19%) patients. A mesh was used in all the cases. We approximated the conjoint tendon to the inguinal ligament to reinforce the floor of the inguinal canal and allow better unfolding of the mesh in 46 (79.3%) patients. 23 (39.7%) patients in the GIH group underwent resection of organ or hernia sac compared to 30 (12.9%) in the control group (p < 0.001). The hernia sac was resected in 20 patients (34.5%) and the omentum was resected in 13 patients (22.4%). Small- or large-bowel resections were not necessary in any case. There was one case (0.34%) of appendix removal in the control group. Drainage was used in 44 (75.9%) patients in the GIH group and in 11 (4.7%) patients in the control group (p < 0.001). The median duration of surgery was 125.5 [102.25–142.5] minutes in the GIH group, which was significantly higher than that in the control group (84 [71–103] minutes, p < 0.001). The median LOS was three days in the GIH group (Table 2).

**Table 2:** Data regarding surgical procedure

		Control Group	GIH Group	P.value
**Number**		**232**	**58**	
Urgent surgery (%)	No	225 (97.0)	51 (87.9)	**0.01**
	Yes	7 (3.0)	7 (12.1)	
Type of anesthesia (%)	General	179(77.2)	44(75.9)	0.862
	Local	53(22.8)	14(24.1)	
Insertion of urinary catheter before surgery		2 (0.9)	11 (19.0)	**< 0.001**
Approximation of Conjoint Tendon to Inguinal Ligament (%)		135 (58.2)	46 (79.3)	**0.004**
Any resection during surgery		30 (12.9)	23 (39.7)	**< 0.001**
Resection of appendix (%)		1 (0.4)	0 (0.0)	1
Resection of omentum (%)		7 (3.0)	13 (22.4)	**< 0.001**
Resection of hernia sac (%)		29 (12.5)	20 (34.5)	**< 0.001**
Use of drainage at surgery		11 (4.7)	44 (75.9)	**< 0.001**
Surgery duration (minutes), [IQR]		84 [71–103]	125.5 [102.25–142.5]	**< 0.001**
Length of stay, median [IQR]		1.00 [1.00–1.00]	3.00 [2.00–4.00]	**< 0.001**

### Outcomes: complications & recurrences

40 [69%] patients in the GIH group had complications compared with 57 (24.6%) patients in the control group (p < 0.001). (Table 3). Patients with GIH had a higher rate of early complications (30 days postoperatively) as inpatients (13.8% vs. 5.6% in the control group, p = 0.045) and outpatients (62.1% vs. 14.7% in the control group, p < 0.001). Patients in the GIH group were at a higher risk of developing more than one complication (6.9% vs. 1.3%, p < 0.001). The most common complication in the GIH group was ischemic orchitis, which was significantly more common than that in the control group (65.5% vs. 13.4%, p < 0.001). Non-surgery-related complications were also more common in the GIH group (6.9% vs. 1.3% in the control group, p = 0.032). There were no cases of abdominal compartment syndrome or deaths after surgery. Two patients (0.7%) had recurrence, both in the control group. The overall follow-up duration was longer for patients in the GIH group (56.00 months [30.00-140.00] in the GIH group vs. 20.50 months [13.00-156.50] in the control group, p = 0.004).

**Table 3:** Description of post-operative complications

		Control Group	GIH Group	p value
**Number**		**232**	**58**	
Length of follow-up, median (IQR)		20.50 [13.00-156.50]	56.00 [30.00-140.00]	**0.004**
Early complication (in hospital) (%)		13 (5.6)	8 (13.8)	**0.045**
Early complication - following discharge (%)		34 (14.7)	36 (62.1)	**< 0.001**
Late complication (> 30 days) (%)		12 (5.2)	1 (1.7)	0.476
Number of early complications (%)	0	185 (79.7)	19 (32.8)	**< 0.001**
	1	44 (19.0)	35 (60.3)	
	2	3 (1.3)	3 (5.2)	
	3	0 (0.0)	1 (1.7)	
Any complication (early, late, recurrence) (%)		57 (24.6)	40 (69.0)	**< 0.001**
Recurrence (%)		2 (0.9)	0 (0.0)	1
Clavien-Dindo grade (%)	1	41 (89.1)	34 (89.5)	0.581
	2	5 (10.9)	3 (7.9)	
	3B	0 (0.0)	1 (2.6)	
Ischemic orchitis (%)		31 ( 13.4)	38 (65.5)	**< 0.001**
Non-surgical related complications (%)		3 (1.3)	4 (6.9)	**0.032**
Seroma (%)		5 (2.2)	0 (0.0)	0.587
Surgical site infection (%)		5 (2.2)	2 (3.4)	0.63
Urinary retention (%)		4 (1.7)	0 (0.0)	0.587
Chronic pain (%)*		11 (4.7)	0 (0.0)	0.129

*Chronic pain - pain that persists for more than 30 days post-surgery

### Type of complication and severity

In both groups (290 patients), 21 patients (7.2%) had early complications during the in-hospital period. Seventy patients (24.1%) had early complications in the 30 postoperative days after discharge. Five patients (1.7%) had complications, both as inpatients and in the early postoperative period after discharge. Thirteen patients (4.5%) experienced late postoperative complications (≥ 30 days postoperatively). Three patients (1%) experienced both early and late complications. 69 patients (23.4%) developed ischemic orchitis. Seven patients (2.4%) had SSI, four of whom required re-hospitalization. Seven (2.4%) patients developed non-surgical complications, including vasovagal events during urination (n = 2), atrial fibrillation (n = 2), lung atelectasis (n = 1), spinal shock after regional anesthesia (n = 1), and fever (n = 1). Most complications (n = 79) were defined as 1 using the Clavien-Dindo scale. Six patients (2%) had complications of severity grade 2, and one (0.34%) patient had 3b complications (requiring reoperation to evacuate a hematoma). There was no difference in the severity of complications between groups (p = 0.581). Seroma, urinary retention, and chronic pain were not observed in patients in the GIH group.

### Factors associated with complications

To evaluate the presence of confounders, we performed univariate and multivariate analyses for both groups. On univariate analysis, we found that GIH (p < 0.001), hernia containing bowel on physical examination (p < 0.001), inability to palpate the testis (p < 0.001), a long-standing hernia (≥ 1 year, p < 0.001), and previous ipsilateral repair (p = 0.017) were associated with the development of complications. Patients with complications were older (mean age 56.6 ± 19.4 years vs. 51.2 ± 19 in patients without complications, p = 0.022) and more obese (median weight of 80[71.5–88.5] kg vs. 75[66.9–83.0] kg in the non-complications group, p = 0.004). A longer duration of surgery was also a significant factor in the development of complications (median of 103[85–131] minutes in patients with complications) vs. median of 84[71–105] minutes in the non-complication group, p < 0.001). Patients with urinary catheters during surgery and use of drainage also had a significantly higher rate of complications (P = 0.012 and P < 0.001, respectively). Organ resection was not associated with the development of any complications.

In a multivariate logistic regression model, we found that a non-palpable testis was a predictor for the development of complications (odds ratio [OR] = 5.2, 95% confidence interval [CI]: 1.6–18.5, P = 0.01). The necessity for drain placement during surgery was also a predictor for the development of complications (OR = 3, 95%CI: 1.04–8.81, P = 0.041).

## Discussion

The aim of this study was to describe and evaluate the treatment of patients with GIH using a mesh through a standard transverse inguinal incision without abdominal cavity preparation. To the best of our knowledge, this report describing 58 patients with GIH is the largest series to date. Our cardinal finding is that GIH can be safely repaired using an open tension-free method without abdominal preparation and/or bowel resection, with acceptable morbidity.

Several interesting points emerge from our study. Several characteristics differentiate between patients with GIH and those with standard hernias. Patients with GIH suffer from their hernia for years, and giant hernias are most often on the left side. They are generally more obese and are more likely to suffer from congestive heart failure. On physical examination, the hernia often contains bowel and the testicle cannot be palpated. In terms of surgery, our operating technique made it possible to treat patients with an acceptable operating time of just over two hours. The average hospitalization duration was 3 days.

GIH is not a common condition, the literature is scarce, and international guidelines for groin hernia management do not outline a treatment strategy for GIH [2]. Case reports describe multiple surgical techniques with or without abdominal cavity preparation [8]. Trakarnsagna et al. reviewed the literature on various case reports between 1986 and 2014. Of these 16 patients, seven (43.8%) required colonic resection. In four cases a surgical technique of abdominal cavity augmentation was used, and in one case preparation of the abdominal cavity was prepared using pneumoperitoneum. The authors recommended routine bowel preparation before surgery [8]. In a previous study using inguinal incision without abdominal preparation, Bierca et al. described the results of elective Lichtenstein GIH repair in 15 patients [9]. In that study, 60% additional resections were performed: omentectomy (n = 7), appendectomy (n = 2), and resection of the testicle and spermatic cord (n = 1). Savoie et al. reported their experiences in a humanitarian mission. They repaired 25 GIH using the Basini approach without mesh and without abdominal preparation [7]. They used a division technique of the hernial sac with the distal sac remaining in the scrotum. Using this technique, they did not describe any need for resection. However, seroma formation and SSI rates were high (12%). In our study, resection of the hernial sac or omentum was performed in 40% of cases, but there was no need for bowel resection. Based on our experience and the literature, we recommend that routine abdominal cavity and bowel preparations are unnecessary in these cases.

With regard to the complication rate, Bierca et al. described an early complication rate of 73%, most commonly postsurgical hematoma. Five patients (33%) required a reoperation. SSI was observed in two cases (13%) [9]. Savoie et al. reported a 12% rate of SSI and a 12% rate of post-operative seroma. We reported that 69% of patients developed complications, but most of them were of low severity. This complication rate is similar to that reported in other studies. In the multivariate model, hernia size alone was not related to a higher risk of complications. However, a non-palpable testis and the requirement for drain placement were predictors of post-operative complications. These findings may be related to the difficulty in reducing the hernia preoperatively (non-palpable testis) and the degree of dissection necessary to reduce the hernia during surgery (requirement for drainage). It is interesting to note the absence of a high SSI rate despite frequent drain use.

Savoie et al. reported a 16% rate of ischemic orchitis after GIH repair. To prevent this complication, some authors recommend routine preventive orchiectomy for GIH surgical treatment [4]. Bierca et al. described a case of preventive orchiectomy but no cases of ischemic orchitis [9]. The most common complication in our study was ischemic orchitis, which occurred in two-thirds of patients in the GIH group. This high number probably reflects the degree of dissection required to reduce the hernia and allow tension-free repair. During the study period, we did not perform a preventive orchiectomy. In most cases of ischemic orchitis, the acute condition subsided post-operatively [2].

We also noted a relatively high percentage of ischemic orchitis in the control group (13.4%) compared with the low percentage (3%) in different prospective studies [10]. Possible explanations include extensive surgical dissection of the hernia sac, especially in inguinoscrotal hernias, and the use of a specific mesh that may compress the testicular vessels. Surgeons repairing large hernias are often faced with the dilemma of ‘to completely dissect the sac or not to completely dissect the sac’. We usually choose the ‘completely dissect the sac’ strategy, taking into account the higher rate of testicular ischemia associated with this approach. We plan a future study to better understand the causes of this finding.

Compared to the literature, we present a relatively high number of patients with GIH without recurrence. Surgical technique and low surgical volume are risk factors for recurrence [2]. In this study, all procedures in both groups were performed by the same expert surgeon, which guaranteed a standardized technique. Our study results, along with Bierca’s and Savoie’s findings, emphasize the meta-analysis of Burcharth, which shows that hernia size itself is not a risk factor for recurrence, even in GIH [11].

This study is not free of limitations. The retrospective data collection and the random selection of the control group could have resulted in selection bias. The control group was composed of different hernia types and sizes (including uncomplicated and inguinoscrotal hernia; direct and indirect hernia). Due to the retrospective nature of this cohort, we could not assess the influence of different hernia types in the control group on the outcomes. The relatively short follow-up period is also a limitation. More studies, preferably prospective studies are needed to define the optimal treatment for patients with GIH.

## Conclusion

Treatment of GIH using an open tension-free method without abdominal preparation is feasible and safe. This technique obviates the need for preliminary or repeated procedures and surgeries. Early complications, mainly ischemic orchitis, are more common than non-GIH repair, but without long-term implications and with a very low recurrence rate.

## Data Availability

The datasets used and/or analyzed during the current study are available from the corresponding author on reasonable request.

## Abbreviations

ASA: American Society of Anesthesiologists
CHF: Congestive heart failure
COPD: Chronic obstructive pulmonary disease
CRF: Chronic renal failure
CVA: Cerebrovascular accident
DM: Diabetes mellitus
HTN: Hypertension
IHD: Ischemic heart disease
PVD: Peripheral vascular disease
VHD: Valvular heart disease
